# Crystal structure of a compact three-dimensional metal–organic framework based on Cs^+^ and (4,5-di­cyano-1,2-phenyl­ene)bis­(phospho­nic acid)

**DOI:** 10.1107/S2056989016016765

**Published:** 2016-11-15

**Authors:** Ricardo F. Mendes, Nutalapati Venkatramaiah, João P. C. Tomé, Filipe A. Almeida Paz

**Affiliations:** a Department of Chemistry, CICECO - Aveiro Institute of Materials, University of Aveiro, 3810-193 Aveiro, Portugal; b Department of Chemistry, QOPNA, University of Aveiro, 3810-193 Aveiro, Portugal; c Centro de Química Estrutural, Instituto Superior Técnico, Universidade de Lisboa, Av. Rovisco Pais, 1049-001 Lisboa, Portugal

**Keywords:** crystal structure, caesium, metal–organic framework, phospho­nic acid ligand

## Abstract

A new metal–organic framework compound, poly[[μ_7_-dihydrogen (4,5-di­cyano-1,2-phenyl­ene)diphospho­nato]­(oxonium)caesium], [Cs(C_8_H_4_N_2_O_6_P_2_)(H_3_O)]_*n*_ (I), based on Cs^+^ and the organic linker 4,5-di­cyano-1,2-phenyl­ene)bis­(phospho­nic acid, (H_4_cpp), containing two distinct coordinating functional groups, has been prepared by a simple diffusion method and its crystal structure is reported. The coordination polymeric structure is based on a CsO_8_N_2_ complex unit comprising a monodentate hydro­nium cation, seven O-atom donors from two phospho­nium groups of the (H_2_cpp)^2−^ ligand, and two N-atom donors from bridging cyano groups. The high level of connectivity from both the metal cation and the organic linker allow the formation of a compact and dense three-dimensional network without any crystallization solvent. Topologically (I) is a seven-connected uninodal network with an overall Schäfli symbol of {4^17^.6^4^}. Metal cations form an undulating inorganic layer, which is linked by strong and highly directional O—H⋯O hydrogen-bonding inter­actions. These metallic layers are, in turn, connected by the organic ligands along the [010] direction to form the overall three-dimensional framework structure.

## Abstract 2

The three-dimensional metal–organic framework compound prepared from Cs^+^ and the organic linker 4,5-di­cyano-1,2-phenyl­ene)bis­(phospho­nic acid is based on an irregular CsO_8_N_2_ coordination center comprising a single monodentate hydro­nium O-atom donor, together with multiple bridging links to the two phospho­nate O-atom donors and to the two nitrile N-atom donors.

## Chemical context   

The area of metal–organic frameworks (MOFs) and coordin­ation polymers (CPs) has proven to be of great importance, not only in academic research but also for industrial applications (Silva *et al.*, 2015 ▸). The simple and easy preparation of these materials, allied with the enormous variety of building blocks (either metal atoms or organic linkers) make these materials ideal to be employed in different applications: gas sorption/separation (Sumida *et al.*, 2012 ▸), as heterogeneous catalysts (Mendes *et al.*, 2015 ▸), luminescence (Heine & Müller-Buschbaum, 2013 ▸), batteries and as corrosion inhibitors (Morozan & Jaouen, 2012 ▸), among many others. Most of these compounds are obtained by mixing transition metal cations with carb­oxy­lic acids. The use of other oxygen-based donor groups such as phospho­nic acids has seen a great resurgence in recent years. The use of mixed oxygen–nitro­gen donor organic linkers is relatively less common, as confirmed by a search of the Cambridge Structural Database (CSD) (Groom *et al.*, 2016 ▸).

Although alkali-metal cations are of great inter­est due to their abundance in biological systems, there is a surprisingly small number of MOFs/CPs based on these elements. Cs^+^-based materials are not as common as other alkali metals, especially when coordinated by either phospho­nic or sulfonic acid residues. Reports on these structures are directed to solely structural descriptions rather than to applications. Nevertheless, these compounds can be used as functional materials in batteries, either as proton conductors (Bazaga-Garcia *et al.*, 2015 ▸) or as insulators (Tominaka *et al.*, 2013 ▸).
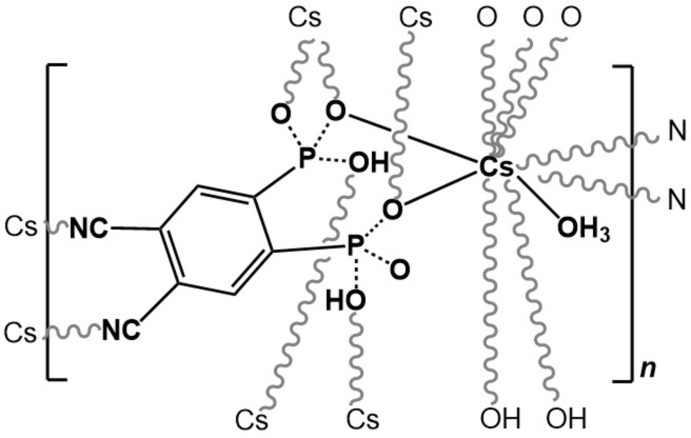


Following our inter­est in this field of research, we report the preparation of a new compact and dense MOF network, [Cs(H_2_cpp)(H_3_O)]_*n*_, prepared by the self-assembly of Cs^+^ and the organic linker (4,5-di­cyano-1,2-phenyl­ene)bis­(phospho­nic acid), (H_4_cpp), previously reported by our group (Venkatramaiah *et al.*, 2015 ▸). The title compound, [Cs(H_2_cpp)(H_3_O)]_*n*_ (I), was assembled under atmospheric conditions and represents, to the best of our knowledge, the first reported MOF or CP based on an amino/cyano phospho­nate with caesium as the metal cation, and the crystal structure is reported herein.

## Structural commentary   

**Figure 1:**
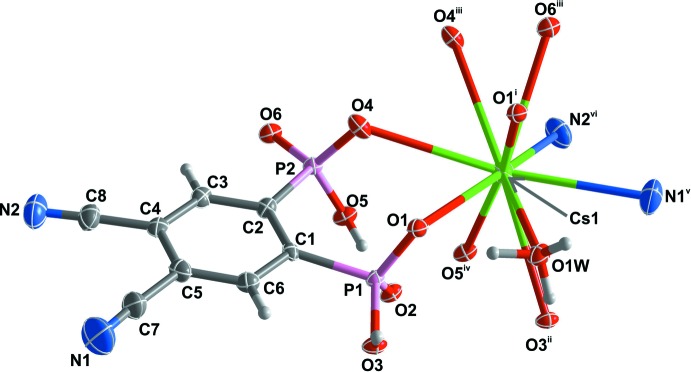
The asymmetric unit of [Cs(H_2_cpp)(H_3_O)]_*n*_ (I) showing all non-hydrogen atoms represented as displacement ellipsoids drawn at the 50% probability level and hydrogen atoms as small spheres with arbitrary radius. The coordination sphere of Cs^+^ is completed by generating (through symmetry) the remaining oxygen and nitro­gen atoms. For symmetry codes, see Table 1 ▸.

The asymmetric unit of (I) comprises one Cs^+^ atom coordinated by a dianionic H_2_cpp^2−^ ligand, together with a monodentate hydro­nium cation (Fig. 1 ▸). The irregular CsO_8_N_2_ coordination polyhedron is defined by the O atom of one monodentate hydronium molecule, six hydrogen phospho­nate O-atom donors and two cyano N-atom donors. The Cs—O bond-length range is 3.159 (4)–3.410 (3) Å and for Cs—N, 3.234 (7) and 3.334 (6) Å (Table 1 ▸). These values are in good agreement with those reported for other phospho­nate-based materials as found in a search in the Cambridge Structural Database (CSD; Groom *et al.*, 2016 ▸): mean value of 3.24 Å for the Cs—O bond (CSD range, 3.01–3.41 Å), and 3.28 Å for the Cs—N bond (CSD range, 2.35–3.79 Å).

**Figure 2:**
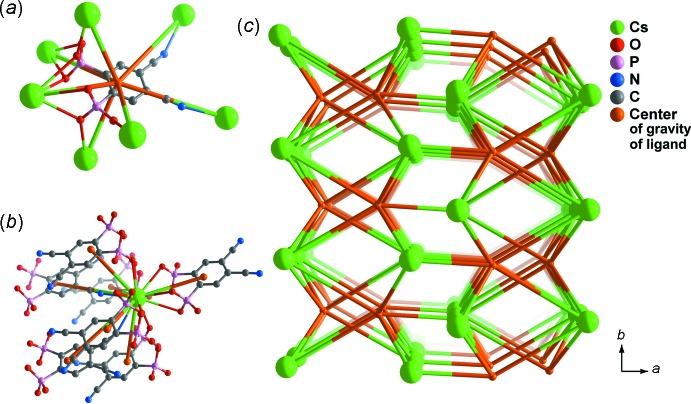
Schematic representation of the connectivity of (*a*) the anionic H_2_cpp^2−^ ligand; (*b*) the Cs^+^ cation and (*c*) the seven-connected [Cs(H_2_cpp)(H_3_O)] uninodal network with an overall Schäfli symbol of {4^17^.6^4^}.

The crystallographic independent H_2_cpp^2−^ residue in (I) acts as a linker connecting seven symmetry-related Cs^+^ metal atoms. The coordination modes between cyano and phospho­nate groups are, as expected, different. While the cyano groups connect to two different metal atoms, each in a simple κ^1^ coordination mode, the two phospho­nate groups coordinate to the remaining metals by κ^1^-*O*, κ^2^-*O* and μ_2_-*O,O* coordination modes. This high coordination of the phospho­nate groups is responsible for the formation of a metallic undulating inorganic layer lying in the *ac* plane of the unit cell. Within this layer, the inter­metallic Cs⋯Cs distances range from 5.7792 (4) to 7.8819 (5) Å (Fig. 2 ▸). The cyano groups are, on the other hand, responsible for the inter-layer connections along the [010] direction. In this case, the inter­metallic Cs⋯Cs distances between layers range from 9.7347 (6) to 9.9044 (6) Å. Although the organic linkers are stacked, the minimum inter-centroid distance of 4.6545 (3) Å (as calculated using *PLATON*: Spek, 2009 ▸) indicates the absence of any significant π–π stacking inter­actions.

The unusual presence of a coordinating H_3_O^+^ ion in this Cs^+^ structure is confirmed by the location of the three hydrogen atoms associated with this cation, which were clearly visible from difference-Fourier maps and by the presence of the double charge with respect to the delocalized P1—O1, P1—O2 and P2—O4, P1—O6 bonds [1.499 (4), 1.509 (3) Å and 1.497 (4), 1.495 (3) Å, respectively]. The P1—O3 and P2—O5 bond lengths for the protonated groups are 1.558 (4) and 1.572 (4) Å, respectively. In addition, although the distance between O1*W* and O4 is very short, suggesting a possible O4—H⋯O1*W* inter­action, a calculated site for such a hydrogen was found to be sterically impossible in the crowded environment about Cs. Not only that, but any attempts to refine this mol­ecule as a coordination water mol­ecule proved to be not as successful as the hydro­nium cation. When the proton is connected to the adjacent phospho­nic residue, the bond is only possible by restraining the O—H distance between O4 and the proton. Also there was still a residual charge near O1*W*, which corroborated the initial refinement.

## Topology   

**Figure 3:**
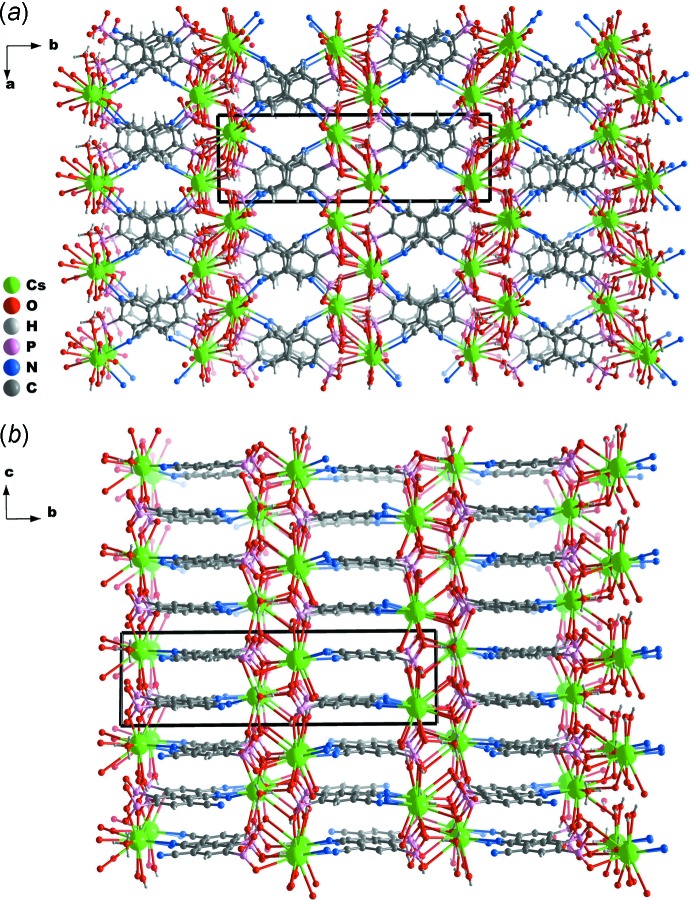
Schematic representation of the crystal packing of [Cs(H_2_cpp) (H_3_O)]_*n*_ viewed in perspective (*a*) along [001] and (*b*) along [100]. The representations emphasize the connection of the undulating inorganic layers located in the *ac* plane of the unit cell (and formed by the metal cations) through the organic ligand. The bottom representation further emphasizes the stacking of the organic linkers with inter-centroid ring distances of 4.6545 (3) Å.

The various coordination modes of the ligand and the presence of a compact undulating inorganic layer formed by the metal atoms to form the MOF architecture can be better understood from a pure topological perspective. Based on the recommendations of Alexandrov *et al.* (2011 ▸), any moiety (ligand, atom or clusters of atoms) connecting more than two metallic centers (μ_*n*_) should be considered as a network node. For (I), all crystallographically independent moieties comprising the asymmetric unit, both the Cs^+^ cation and the anionic H_2_cpp^2−^ ligand, should therefore be considered as nodes. Using the software package *TOPOS* (Blatov & Shevchenko, 2006 ▸), (I) could be classified as a seven-connected uninodal network with an overall Schäfli symbol of {4^17^.6^4^}. Fig. 2 ▸ illustrates the breakdown of the network of (I) into nodes and connecting rods, with the individual connectivity of each node being superimposed into the crystal structure itself (Fig. 2 ▸ *a* and 2*b*). The metal atom and the organic linker are connected to each other in every direction of the unit cell (Fig. 2 ▸ *c*), forming a compact and robust three-dimensional network (Fig. 3 ▸). The absence of water mol­ecules of crystallization leads to this very compact structure having no solvent-accessible pores: only 0.2% of the unit cell volume [calculated using *Mercury* (Macrae *et al.*, 2006 ▸)] corresponds to voids.

## Supra­molecular features   

**Figure 4:**
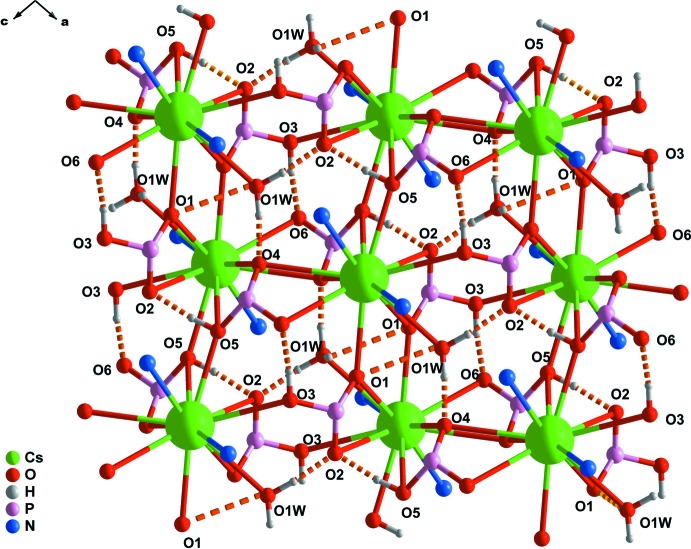
Schematic representation of a portion of the undulating inorganic layer comprising the crystal structure of (I), emphasizing the various strong and directional supra­molecular O—H⋯O hydrogen-bonding inter­actions (orange dashed lines) present within this layer. For geometrical details and symmetry codes, see Table 2 ▸.

The lack of crystallization solvent mol­ecules in (I) results in a rather small number of crystallographically different hydrogen-bonding supra­molecular inter­actions (Table 2 ▸). Indeed, although the structure is rich in hydrogen-bonding acceptors, only the POH and the H_3_O^+^ moieties can establish strong inter­actions. A total of five distinct hydrogen bonds are present, two of these involving the phospho­nic acid donor groups [O3—H3⋯O6^vii^ and O5—H5⋯O2) and three involving the H_3_O^+^ moiety (O1*W*—H1*X*⋯O2^iv^, O1*W*—H1*Y*⋯O1 and O1*W*—H1*Z*⋯O4^iii^ (for symmetry codes, see Tables 1 ▸ and 2 ▸)]. An overall three-dimensional network structure is generated in which there are 62 Å^3^ voids (though not solvent-accessible ones). No π–π ring inter­actions are present (minimum ring-centroid separation = 4.655 Å). These hydrogen bonds are confined within the inorganic undulating layer (Fig. 4 ▸).

## Database survey   

Although unusual in the case of Cs, in the Cambridge Structural Database (CSD) a total of 45 structures in which coordination between the metal cation and the hydro­nium cation is present, *e.g*. among the metal complexes (Reyes-Martínez *et al.*, 2009 ▸; Jennifer *et al.*, 2014 ▸; Teng *et al.*, 2016 ▸; Hu & Mak, 2013 ▸) and coordination polymer/metal–organic frameworks (Yotnoi *et al.*, 2015 ▸; Wang *et al.*, 2013 ▸; Humphrey *et al.*, 2005 ▸). Wang *et al.* (2013 ▸) in fact reported the structures of an isotypic series of crystal materials involving lanthanides (Nd, Sm, Eu, Gd, Tb, Dy, Ho, Er and Y), in which the presence of the coordinating hydro­nium cation was confirmed.

## Synthesis and crystallization   

Chemicals were purchased from commercial sources and used without any further purification steps. (4,5-Di­cyano-1,2-phenyl­ene)bis­(phospho­nic acid) (H_4_cpp) was prepared according to published procedures (Venkatramaiah *et al.*, 2015 ▸).

Synthesis of [Cs(H_2_cpp)(H_3_O)]_*n*_, (I): H_4_cpp (29 mg, 0.1 m*M*) was dissolved in 4 ml of methanol. A 1 ml aliquot of a methano­lic caesium hydroxide solution (45 mg, 0.3 m*M*; Sigma Aldrich, puriss p.a. ≥ 96%) was added slowly. The resulting mixture was stirred at ambient temperature for 10 min for uniform mixing. The final solution was allowed to slowly evaporate at ambient temperature. White transparent crystals of the title compound were obtained after one week. Crystals were filtered and dried under vacuum.

## Refinement   

Crystal data, data collection and structure refinement details are summarized in Table 3 ▸. Hydrogen atoms bound to carbon were placed at idealized positions with C—H = 0.95 Å and included in the final structural model in a riding-motion approximation with the isotropic displacement parameters fixed at 1.2*U*_eq_(C). Hydrogen atoms associated with the H_3_O^+^ moiety and the phospho­nate groups were clearly located from difference-Fourier maps and were included in the refinement with the O—H and H⋯H (only for the cation) distances restrained to 0.95 (1) and 1.55 (1) Å, respectively, in order to ensure a chemically reasonable environment for these moieties. These hydrogen atoms were modelled with the isotropic displacement parameters fixed at 1.5*U*_eq_(O). In order to avoid a close proximity between the H atoms associated with the POH group and the H_3_O^+^ cation and the central Cs^+^ ion in the crystal structure, an anti­bump restraint [3.5 (1) Å)] was included in the overall refinement.

## Supplementary Material

Crystal structure: contains datablock(s) I, New_Global_Publ_Block. DOI: 10.1107/S2056989016016765/zs2366sup1.cif

Structure factors: contains datablock(s) I. DOI: 10.1107/S2056989016016765/zs2366Isup2.hkl

CCDC reference: 1510674

Additional supporting information: crystallographic information; 3D view; checkCIF report

## Figures and Tables

**Table 1:** Selected bond lengths (Å)

Cs1—O1	3.400 (3)	Cs1—O6^v^	3.259 (4)
Cs1—O1*W*	3.388 (4)	Cs1—O5^vi^	3.159 (4)
Cs1—O4	3.269 (4)	P1—O1	1.499 (4)
Cs1—N1^i^	3.234 (7)	P1—O2	1.509 (4)
Cs1—N2^ii^	3.334 (6)	P1—O3	1.558 (4)
Cs1—O1^iii^	3.229 (3)	P2—O4	1.497 (4)
Cs1—O3^iv^	3.356 (4)	P2—O5	1.572 (4)
Cs1—O4^v^	3.410 (3)	P2—O6	1.495 (3)

Symmetry codes: (i) 
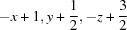
; (ii) 
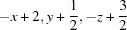
; (iii) 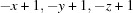; (iv) 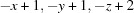; (v) 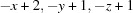; (vi) 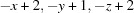.

**Table 2:** Hydrogen-bond geometry (Å, °)

*D*—H⋯*A*	*D*—H	H⋯*A*	*D*⋯*A*	*D*—H⋯*A*
O3—H3⋯O6^vii^	0.95 (1)	1.59 (12)	2.528 (5)	172 (5)
O5—H5⋯O2	0.94 (1)	1.60 (12)	2.545 (5)	175 (5)
O1*W*—H1*X*⋯O2^iv^	0.95 (1)	1.64 (16)	2.553 (5)	160 (4)
O1*W*—H1*Y*⋯O1	0.96 (1)	1.66 (11)	2.526 (5)	149 (4)
O1*W*—H1*Z*⋯O4^iii^	0.95 (1)	1.56 (15)	2.485 (5)	162 (4)

Symmetry codes: (iii) 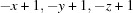; (iv) 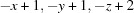; (vii) 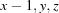.

**Table 3:** Experimental details

Crystal data	
Chemical formula	[Cs(C_8_H_4_N_2_O_6_P_2_)(H_3_O)]
*M*_r_	438.01
Crystal system, space group	Monoclinic, *P*2_1_/*c*
Temperature (K)	180
*a*, *b*, *c* (Å)	7.8819 (5), 24.5497 (14), 7.3137 (4)
β (°)	98.739 (2)
*V* (Å^3^)	1398.76 (14)
*Z*	4
Radiation type	Mo *K*α
μ (mm^−1^)	2.91
Crystal size (mm)	0.15 × 0.06 × 0.02

Data collection	
Diffractometer	Bruker D8 QUEST
Absorption correction	Multi-scan (*SADABS*; Bruker 2012 ▸)
*T*_min_, *T*_max_	0.647, 0.747
No. of measured, independent and observed [*I* > 2σ(*I*)] reflections	27787, 2550, 2499
*R*_int_	0.021
(sin θ/λ)_max_ (Å^−1^)	0.602

Refinement	
*R*[*F* ^2^ > 2σ(*F* ^2^)], *wR*(*F* ^2^), *S*	0.031, 0.080, 1.50
No. of reflections	2550
No. of parameters	196
No. of restraints	10
H-atom treatment	H atoms treated by a mixture of independent and constrained refinement
Δρ_max_, Δρ_min_ (e Å^−3^)	0.70, −0.60

Computer programs: *APEX2* and *SAINT* (Bruker, 2012 ▸), *SHELXS* (Sheldrick, 2008 ▸), *SHELXL2014* (Sheldrick, 2015 ▸) and *DIAMOND* (Brandenburg, 1999 ▸).

## supplementary crystallographic information

### Crystal data

**Table:**

[Cs(C_8_H_4_N_2_O_6_P_2_)(H_3_O)]	*F*(000) = 840
*M**_r_* = 438.01	*D*_x_ = 2.080 Mg m^−^^3^
Monoclinic, *P*2_1_/*c*	Mo *K*α radiation, λ = 0.71073 Å
*a* = 7.8819 (5) Å	Cell parameters from 9290 reflections
*b* = 24.5497 (14) Å	θ = 2.7–36.7°
*c* = 7.3137 (4) Å	µ = 2.91 mm^−^^1^
β = 98.739 (2)°	*T* = 180 K
*V* = 1398.76 (14) Å^3^	Plate, colourless
*Z* = 4	0.15 × 0.06 × 0.02 mm

### Data collection

**Table:**

Bruker D8 QUEST diffractometer	2550 independent reflections
Radiation source: Sealed tube	2499 reflections with *I* > 2σ(*I*)
Multi-layer X-ray mirror monochromator	*R*_int_ = 0.021
Detector resolution: 10.4167 pixels mm^-1^	θ_max_ = 25.4°, θ_min_ = 3.6°
ω/φ scans	*h* = −9→9
Absorption correction: multi-scan (SADABS; Bruker 2012)	*k* = −29→29
*T*_min_ = 0.647, *T*_max_ = 0.747	*l* = −8→8
27787 measured reflections	

### Refinement

**Table:**

Refinement on *F*^2^	10 restraints
Least-squares matrix: full	Hydrogen site location: mixed
*R*[*F*^2^ > 2σ(*F*^2^)] = 0.031	H atoms treated by a mixture of independent and constrained refinement
*wR*(*F*^2^) = 0.080	*w* = 1/[σ^2^(*F*_o_^2^) + (0.0134*P*)^2^ + 6.7796*P*] where *P* = (*F*_o_^2^ + 2*F*_c_^2^)/3
*S* = 1.50	(Δ/σ)_max_ = 0.002
2550 reflections	Δρ_max_ = 0.70 e Å^−^^3^
196 parameters	Δρ_min_ = −0.60 e Å^−^^3^

### Special details

**Table:**

Geometry. All esds (except the esd in the dihedral angle between two l.s. planes) are estimated using the full covariance matrix. The cell esds are taken into account individually in the estimation of esds in distances, angles and torsion angles; correlations between esds in cell parameters are only used when they are defined by crystal symmetry. An approximate (isotropic) treatment of cell esds is used for estimating esds involving l.s. planes.

### Fractional atomic coordinates and isotropic or equivalent isotropic displacement parameters (Å^2^)

**Table:**

	*x*	*y*	*z*	*U*_iso_*/*U*_eq_	
Cs1	0.78119 (4)	0.56625 (2)	0.69290 (4)	0.02080 (11)	
O1W	0.3543 (5)	0.54753 (15)	0.6850 (5)	0.0224 (8)	
H1X	0.330 (6)	0.5549 (18)	0.806 (3)	0.034*	
H1Y	0.3732 (16)	0.5092 (4)	0.675 (6)	0.034*	
H1Z	0.258 (4)	0.5572 (15)	0.597 (5)	0.034*	
P1	0.56069 (15)	0.42656 (5)	0.83441 (16)	0.0139 (2)	
P2	0.97399 (15)	0.40970 (5)	0.72111 (17)	0.0147 (3)	
O1	0.5083 (4)	0.45821 (14)	0.6597 (5)	0.0197 (7)	
O2	0.6914 (4)	0.45406 (14)	0.9768 (5)	0.0186 (7)	
O3	0.4048 (4)	0.41059 (15)	0.9310 (5)	0.0185 (7)	
H3	0.311 (5)	0.403 (2)	0.837 (6)	0.028*	
O4	0.8955 (4)	0.44573 (15)	0.5659 (5)	0.0237 (8)	
O5	0.9966 (4)	0.44125 (15)	0.9103 (5)	0.0217 (8)	
H5	0.886 (3)	0.4463 (14)	0.943 (7)	0.033*	
O6	1.1438 (4)	0.38507 (14)	0.7016 (5)	0.0203 (7)	
N1	0.4101 (9)	0.1823 (3)	0.7906 (11)	0.0603 (19)	
N2	0.9099 (8)	0.1597 (2)	0.7224 (8)	0.0435 (14)	
C1	0.6536 (6)	0.36144 (19)	0.7793 (6)	0.0143 (9)	
C2	0.8230 (6)	0.3543 (2)	0.7429 (6)	0.0160 (10)	
C3	0.8843 (6)	0.3016 (2)	0.7237 (7)	0.0189 (10)	
H3A	0.9990	0.2968	0.7014	0.023*	
C4	0.7834 (7)	0.2561 (2)	0.7360 (6)	0.0201 (10)	
C5	0.6129 (7)	0.2632 (2)	0.7663 (7)	0.0222 (11)	
C6	0.5506 (6)	0.3153 (2)	0.7867 (7)	0.0194 (10)	
H6A	0.4350	0.3198	0.8061	0.023*	
C7	0.5014 (8)	0.2170 (2)	0.7777 (9)	0.0333 (14)	
C8	0.8522 (8)	0.2021 (2)	0.7244 (8)	0.0294 (12)	

### Atomic displacement parameters (Å^2^)

**Table:**

	*U*^11^	*U*^22^	*U*^33^	*U*^12^	*U*^13^	*U*^23^
Cs1	0.02062 (17)	0.02091 (18)	0.02049 (17)	−0.00137 (12)	0.00196 (12)	−0.00099 (12)
O1W	0.0225 (18)	0.0260 (19)	0.0182 (18)	0.0044 (15)	0.0015 (15)	−0.0030 (15)
P1	0.0117 (6)	0.0164 (6)	0.0135 (6)	0.0012 (5)	0.0021 (4)	−0.0005 (5)
P2	0.0109 (6)	0.0189 (6)	0.0142 (6)	−0.0009 (5)	0.0014 (5)	−0.0007 (5)
O1	0.0225 (18)	0.0222 (18)	0.0151 (17)	0.0047 (14)	0.0051 (14)	0.0011 (14)
O2	0.0139 (16)	0.0240 (18)	0.0177 (17)	0.0003 (14)	0.0020 (13)	−0.0046 (14)
O3	0.0126 (16)	0.0276 (19)	0.0156 (17)	0.0001 (14)	0.0034 (13)	−0.0003 (14)
O4	0.0198 (18)	0.0239 (19)	0.0257 (19)	−0.0040 (15)	−0.0019 (15)	0.0079 (16)
O5	0.0160 (17)	0.028 (2)	0.0216 (18)	−0.0040 (14)	0.0041 (14)	−0.0063 (15)
O6	0.0136 (17)	0.0279 (19)	0.0187 (18)	0.0005 (14)	0.0003 (14)	−0.0020 (15)
N1	0.067 (4)	0.037 (3)	0.082 (5)	−0.024 (3)	0.029 (4)	−0.006 (3)
N2	0.054 (3)	0.029 (3)	0.045 (3)	0.012 (3)	0.000 (3)	−0.003 (2)
C1	0.018 (2)	0.014 (2)	0.010 (2)	0.0009 (18)	−0.0006 (18)	0.0020 (18)
C2	0.013 (2)	0.018 (2)	0.015 (2)	−0.0004 (19)	−0.0017 (18)	−0.0024 (19)
C3	0.017 (2)	0.023 (3)	0.015 (2)	0.005 (2)	−0.0022 (19)	0.000 (2)
C4	0.029 (3)	0.021 (3)	0.008 (2)	0.004 (2)	−0.002 (2)	−0.0014 (19)
C5	0.026 (3)	0.020 (3)	0.020 (3)	−0.006 (2)	0.003 (2)	−0.002 (2)
C6	0.017 (2)	0.022 (3)	0.019 (3)	−0.002 (2)	0.0028 (19)	−0.001 (2)
C7	0.040 (3)	0.023 (3)	0.039 (4)	−0.006 (3)	0.015 (3)	−0.004 (3)
C8	0.037 (3)	0.024 (3)	0.026 (3)	0.002 (2)	0.002 (2)	−0.004 (2)

### Geometric parameters (Å, º)

**Table:**

Cs1—O1	3.400 (3)	O1W—H1X	0.95 (3)
Cs1—O1W	3.388 (4)	O1W—H1Y	0.957 (11)
Cs1—O4	3.269 (4)	O1W—H1Z	0.95 (3)
Cs1—N1^i^	3.234 (7)	O3—H3	0.95 (4)
Cs1—N2^ii^	3.334 (6)	O5—H5	0.95 (3)
Cs1—O1^iii^	3.229 (3)	N1—C7	1.128 (9)
Cs1—O3^iv^	3.356 (4)	N2—C8	1.137 (7)
Cs1—O4^v^	3.410 (3)	C1—C2	1.412 (7)
Cs1—O6^v^	3.259 (4)	C1—C6	1.399 (7)
Cs1—O5^vi^	3.159 (4)	C2—C3	1.396 (7)
P1—O1	1.499 (4)	C3—C4	1.382 (7)
P1—O2	1.509 (4)	C4—C8	1.440 (7)
P1—O3	1.558 (4)	C4—C5	1.406 (8)
P1—C1	1.829 (5)	C5—C7	1.445 (8)
P2—O4	1.497 (4)	C5—C6	1.386 (7)
P2—O5	1.572 (4)	C3—H3A	0.9500
P2—O6	1.495 (3)	C6—H6A	0.9500
P2—C2	1.830 (5)		
			
O1—Cs1—O1W	43.71 (8)	O2—P1—C1	106.8 (2)
O1—Cs1—O4	58.15 (8)	O3—P1—C1	104.5 (2)
O1—Cs1—N1^i^	113.35 (14)	O4—P2—O5	110.8 (2)
O1—Cs1—N2^ii^	170.46 (11)	O4—P2—O6	116.1 (2)
O1—Cs1—O1^iii^	55.63 (9)	O4—P2—C2	107.8 (2)
O1—Cs1—O3^iv^	80.81 (9)	O5—P2—O6	107.5 (2)
O1—Cs1—O4^v^	114.27 (8)	O5—P2—C2	106.1 (2)
O1—Cs1—O6^v^	114.88 (9)	O6—P2—C2	108.1 (2)
O1—Cs1—O5^vi^	106.05 (9)	Cs1—O1—P1	104.72 (16)
O1W—Cs1—O4	100.85 (9)	Cs1—O1—Cs1^iii^	124.37 (11)
O1W—Cs1—N1^i^	69.68 (14)	Cs1^iii^—O1—P1	130.85 (18)
O1W—Cs1—N2^ii^	142.36 (12)	Cs1^iv^—O3—P1	143.32 (19)
O1^iii^—Cs1—O1W	51.81 (9)	Cs1—O4—P2	114.80 (18)
O1W—Cs1—O3^iv^	58.71 (8)	Cs1—O4—Cs1^v^	119.82 (11)
O1W—Cs1—O4^v^	143.40 (9)	Cs1^v^—O4—P2	96.45 (15)
O1W—Cs1—O6^v^	110.36 (8)	Cs1^vi^—O5—P2	138.83 (19)
O1W—Cs1—O5^vi^	114.73 (9)	Cs1^v^—O6—P2	102.81 (17)
O4—Cs1—N1^i^	163.38 (15)	H1X—O1W—H1Z	109 (3)
O4—Cs1—N2^ii^	116.78 (11)	H1Y—O1W—H1Z	108 (3)
O1^iii^—Cs1—O4	78.26 (9)	Cs1—O1W—H1Z	132 (2)
O3^iv^—Cs1—O4	124.05 (9)	Cs1—O1W—H1X	107 (3)
O4—Cs1—O4^v^	60.18 (9)	Cs1—O1W—H1Y	88.2 (9)
O4—Cs1—O6^v^	89.17 (9)	H1X—O1W—H1Y	108 (4)
O4—Cs1—O5^vi^	94.02 (9)	P1—O3—H3	108 (3)
N1^i^—Cs1—N2^ii^	73.64 (16)	Cs1^iv^—O3—H3	104 (3)
O1^iii^—Cs1—N1^i^	85.23 (15)	P2—O5—H5	108 (3)
O3^iv^—Cs1—N1^i^	63.64 (15)	Cs1^vi^—O5—H5	100 (3)
O4^v^—Cs1—N1^i^	119.25 (15)	Cs1^vii^—N1—C7	167.2 (6)
O6^v^—Cs1—N1^i^	81.85 (15)	Cs1^viii^—N2—C8	155.5 (5)
O5^vi^—Cs1—N1^i^	102.33 (15)	P1—C1—C2	124.9 (4)
O1^iii^—Cs1—N2^ii^	133.14 (12)	C2—C1—C6	118.5 (4)
O3^iv^—Cs1—N2^ii^	97.40 (12)	P1—C1—C6	116.4 (4)
O4^v^—Cs1—N2^ii^	64.93 (12)	P2—C2—C3	116.1 (4)
O6^v^—Cs1—N2^ii^	71.74 (12)	P2—C2—C1	124.8 (4)
O5^vi^—Cs1—N2^ii^	65.30 (12)	C1—C2—C3	119.1 (4)
O1^iii^—Cs1—O3^iv^	110.02 (8)	C2—C3—C4	122.1 (5)
O1^iii^—Cs1—O4^v^	92.16 (8)	C3—C4—C5	118.9 (5)
O1^iii^—Cs1—O6^v^	64.05 (8)	C5—C4—C8	120.1 (5)
O1^iii^—Cs1—O5^vi^	161.55 (9)	C3—C4—C8	121.0 (5)
O3^iv^—Cs1—O4^v^	157.79 (8)	C6—C5—C7	119.3 (5)
O3^iv^—Cs1—O6^v^	145.49 (9)	C4—C5—C7	121.1 (5)
O3^iv^—Cs1—O5^vi^	60.47 (8)	C4—C5—C6	119.6 (5)
O4^v^—Cs1—O6^v^	44.67 (8)	C1—C6—C5	121.7 (5)
O4^v^—Cs1—O5^vi^	98.52 (8)	N1—C7—C5	177.0 (7)
O5^vi^—Cs1—O6^v^	133.23 (8)	N2—C8—C4	177.2 (6)
O1—P1—O2	115.3 (2)	C2—C3—H3A	119.00
O1—P1—O3	112.52 (19)	C4—C3—H3A	119.00
O1—P1—C1	109.5 (2)	C1—C6—H6A	119.00
O2—P1—O3	107.6 (2)	C5—C6—H6A	119.00
			
O1W—Cs1—O1—P1	−114.0 (2)	O1—Cs1—O5^vi^—P2^vi^	−110.8 (3)
O1W—Cs1—O1—Cs1^iii^	68.38 (14)	O1W—Cs1—O5^vi^—P2^vi^	−64.9 (3)
O4—Cs1—O1—P1	79.83 (17)	O4—Cs1—O5^vi^—P2^vi^	−168.8 (3)
O4—Cs1—O1—Cs1^iii^	−97.75 (14)	O1—P1—C1—C2	79.5 (4)
N1^i^—Cs1—O1—P1	−116.3 (2)	O1—P1—C1—C6	−105.0 (4)
N1^i^—Cs1—O1—Cs1^iii^	66.1 (2)	O2—P1—C1—C2	−46.0 (4)
O1^iii^—Cs1—O1—P1	177.6 (2)	O2—P1—C1—C6	129.6 (4)
O1^iii^—Cs1—O1—Cs1^iii^	−0.02 (14)	O3—P1—C1—C2	−159.8 (4)
O3^iv^—Cs1—O1—P1	−60.36 (16)	O3—P1—C1—C6	15.7 (4)
O3^iv^—Cs1—O1—Cs1^iii^	122.06 (13)	O3—P1—O1—Cs1	132.24 (17)
O4^v^—Cs1—O1—P1	102.56 (17)	C1—P1—O1—Cs1	−112.09 (19)
O4^v^—Cs1—O1—Cs1^iii^	−75.03 (14)	O2—P1—O1—Cs1^iii^	−174.33 (19)
O6^v^—Cs1—O1—P1	151.95 (15)	O3—P1—O1—Cs1^iii^	−50.4 (3)
O6^v^—Cs1—O1—Cs1^iii^	−25.63 (15)	C1—P1—O1—Cs1^iii^	65.3 (3)
O5^vi^—Cs1—O1—P1	−4.83 (18)	O1—P1—O3—Cs1^iv^	−111.9 (3)
O5^vi^—Cs1—O1—Cs1^iii^	177.59 (11)	O2—P1—O1—Cs1	8.3 (2)
O1—Cs1—O4—P2	−89.90 (19)	C1—P1—O3—Cs1^iv^	129.5 (3)
O1—Cs1—O4—Cs1^v^	156.05 (16)	O2—P1—O3—Cs1^iv^	16.2 (4)
O1W—Cs1—O4—P2	−99.61 (18)	O4—P2—C2—C3	121.5 (4)
O1W—Cs1—O4—Cs1^v^	146.34 (11)	O5—P2—C2—C1	59.4 (4)
N2^ii^—Cs1—O4—P2	80.8 (2)	O6—P2—C2—C1	174.4 (4)
N2^ii^—Cs1—O4—Cs1^v^	−33.23 (17)	O6—P2—C2—C3	−4.7 (4)
O1^iii^—Cs1—O4—P2	−146.54 (19)	O5—P2—C2—C3	−119.8 (4)
O1^iii^—Cs1—O4—Cs1^v^	99.41 (12)	O4—P2—C2—C1	−59.4 (4)
O3^iv^—Cs1—O4—P2	−40.2 (2)	O5—P2—O4—Cs1	−4.8 (2)
O3^iv^—Cs1—O4—Cs1^v^	−154.24 (10)	O6—P2—O4—Cs1	−127.67 (19)
O4^v^—Cs1—O4—P2	114.1 (2)	C2—P2—O4—Cs1	110.9 (2)
O4^v^—Cs1—O4—Cs1^v^	−0.02 (9)	O5—P2—O4—Cs1^v^	122.33 (16)
O6^v^—Cs1—O4—P2	149.81 (18)	O6—P2—O4—Cs1^v^	−0.5 (2)
O6^v^—Cs1—O4—Cs1^v^	35.76 (12)	C2—P2—O4—Cs1^v^	−121.95 (17)
O5^vi^—Cs1—O4—P2	16.53 (19)	O4—P2—O5—Cs1^vi^	−158.3 (2)
O5^vi^—Cs1—O4—Cs1^v^	−97.52 (12)	O6—P2—O5—Cs1^vi^	−30.5 (3)
O1W—Cs1—N2^ii^—C8^ii^	−38.5 (13)	C2—P2—O5—Cs1^vi^	85.0 (3)
O4—Cs1—N2^ii^—C8^ii^	140.8 (11)	O4—P2—O6—Cs1^v^	0.6 (2)
O1—Cs1—O1^iii^—Cs1^iii^	0.00 (10)	O5—P2—O6—Cs1^v^	−124.03 (17)
O1—Cs1—O1^iii^—P1^iii^	176.9 (3)	C2—P2—O6—Cs1^v^	121.81 (17)
O1W—Cs1—O1^iii^—Cs1^iii^	−54.81 (12)	P1—C1—C2—P2	−6.5 (6)
O1W—Cs1—O1^iii^—P1^iii^	122.1 (3)	P1—C1—C2—C3	172.7 (4)
O4—Cs1—O1^iii^—Cs1^iii^	59.28 (12)	C6—C1—C2—P2	178.1 (4)
O4—Cs1—O1^iii^—P1^iii^	−123.8 (2)	C6—C1—C2—C3	−2.8 (7)
O1—Cs1—O3^iv^—P1^iv^	−14.9 (3)	P1—C1—C6—C5	−173.3 (4)
O1W—Cs1—O3^iv^—P1^iv^	25.8 (3)	C2—C1—C6—C5	2.5 (7)
O4—Cs1—O3^iv^—P1^iv^	−55.9 (3)	P2—C2—C3—C4	−179.6 (4)
O1—Cs1—O4^v^—Cs1^v^	−22.22 (15)	C1—C2—C3—C4	1.2 (7)
O1—Cs1—O4^v^—P2^v^	101.24 (17)	C2—C3—C4—C5	0.9 (7)
O1W—Cs1—O4^v^—Cs1^v^	−65.93 (19)	C2—C3—C4—C8	−177.0 (5)
O1W—Cs1—O4^v^—P2^v^	57.5 (2)	C3—C4—C5—C6	−1.2 (7)
O4—Cs1—O4^v^—Cs1^v^	0.00 (10)	C3—C4—C5—C7	179.1 (5)
O4—Cs1—O4^v^—P2^v^	123.46 (19)	C8—C4—C5—C6	176.7 (5)
O1—Cs1—O6^v^—P2^v^	−99.78 (17)	C8—C4—C5—C7	−2.9 (7)
O1W—Cs1—O6^v^—P2^v^	−147.10 (15)	C4—C5—C6—C1	−0.5 (7)
O4—Cs1—O6^v^—P2^v^	−45.83 (17)	C7—C5—C6—C1	179.2 (5)

Symmetry codes: (i) −*x*+1, *y*+1/2, −*z*+3/2; (ii) −*x*+2, *y*+1/2, −*z*+3/2; (iii) −*x*+1, −*y*+1, −*z*+1; (iv) −*x*+1, −*y*+1, −*z*+2; (v) −*x*+2, −*y*+1, −*z*+1; (vi) −*x*+2, −*y*+1, −*z*+2; (vii) −*x*+1, *y*−1/2, −*z*+3/2; (viii) −*x*+2, *y*−1/2, −*z*+3/2.

### Hydrogen-bond geometry (Å, º)

**Table:**

*D*—H···*A*	*D*—H	H···*A*	*D*···*A*	*D*—H···*A*
O3—H3···O6^ix^	0.95 (1)	1.59 (12)	2.528 (5)	172 (5)
O5—H5···O2	0.94 (1)	1.60 (12)	2.545 (5)	175 (5)
O1*W*—H1*X*···O2^iv^	0.95 (1)	1.64 (16)	2.553 (5)	160 (4)
O1*W*—H1*Y*···O1	0.96 (1)	1.66 (11)	2.526 (5)	149 (4)
O1*W*—H1*Z*···O4^iii^	0.95 (1)	1.56 (15)	2.485 (5)	162 (4)

Symmetry codes: (iii) −*x*+1, −*y*+1, −*z*+1; (iv) −*x*+1, −*y*+1, −*z*+2; (ix) *x*−1, *y*, *z*.
